# Building Research Capacity in Africa: Equity and Global Health Collaborations

**DOI:** 10.1371/journal.pmed.1001612

**Published:** 2014-03-11

**Authors:** Kathryn M. Chu, Sudha Jayaraman, Patrick Kyamanywa, Georges Ntakiyiruta

**Affiliations:** 1 Center for Surgery and Public Health, Brigham and Women's Hospital, Harvard Medical School, Department of Surgery, Boston, Massachusetts, United States of America; 2 Virginia Commonwealth University Medical Center, Department of Surgery, Richmond, Virginia, United States of America; 3 University of Rwanda, School of Medicine, Department of Surgery, Butare, Rwanda

## Abstract

Kathryn Chu and colleagues discuss the impact of high-income country investigators conducting research in low- and middle-income countries and explore lessons from the effective and equitable relationships that exist.

*Please see later in the article for the Editors' Summary*

**Summary Points**

- Global health has increased the number of high-income country (HIC) investigators conducting research in low- and middle-income countries (LMICs).
- Partnerships with local collaborators rather than extractive research are needed.
- LMICs have to take an active role in leading or directing these research collaborations in order to maximize the benefits and minimize the harm of inherently inequitable relationships.
- This essay explores lessons from effective and equitable relationships that exist between African countries and HICs.

## Introduction

Global health is a growing academic field where high-income country (HIC) faculty and students work in low- and middle-income countries (LMICs), especially in Africa; learn about new cultures, settings, and diseases; and possibly develop an expertise to address existing and emerging challenges in health care [1]. Global health has brought beneficial HIC medical knowledge particularly to African countries: expertise in health policy and planning from high-income settings has improved clinic and hospital infrastructure and practices such as neonatal resuscitation [2],[3]. In addition, research led and supported by HIC researchers has clearly identified preventive and therapeutic interventions for major causes of mortality such as severe malaria, HIV/AIDS, and childhood sepsis [4]–[7].

Worldwide, the highest burden of disease is from LMICs; however, medical research originating from these countries is low [8]. According to one study, sub-Saharan Africa (SSA) produces less than 1% of biomedical publications [9]. Effective research has four pre-requisites: individual research skills and ability, appropriate infrastructure, relevance to national policies, and the ability to contribute to global research and policy needs [10]. African research capacity has not paralleled capacity in HIC for many reasons: few qualified researchers, less funding, poor infrastructure such as laboratories and computers, and lack of expertise in preparing manuscripts for publication [8]. Collaboration with HIC colleagues and institutions has enormous promise to bring expertise, funding, and resources to Africa. However, there is great potential for a power imbalance in these relationships. Much of the research carried out in Africa is led, funded, and published by HIC researchers without equal collaboration from LMIC colleagues. HIC scientists have been accused of extractive research, flying into an LMIC to obtain data or samples and leaving with the recognition and benefits of the publication. Researchers collecting blood samples for studies have been termed “mosquitoes” or “vampires” [11],[12]. HIC investigators secure most of the funding for global health research projects and often dictate the research agenda [11]. If their values and objectives are different from African partners this can lead to inappropriate projects unrelated to local research needs, and derive conclusions that do not have any direct local benefit [13]. Some participants have commented that these kinds of collaborations leave locals feeling like “prostitutes” [14]. Furthermore, when HIC researchers conduct studies in settings that are unprepared in terms of infrastructure and health workers, research can disrupt local medical and educational services and have a detrimental effect on local health care, usually by taking already overworked health care providers away from their clinical and teaching duties [11],[14],[15].

HIC academics work for universities that typically measure the success of their faculty by research funding and publications. Even if HIC scientists genuinely want to advance African research agendas, building the research capacity of African collaborators may not be an important objective to their institutions [13],[14].

## The Challenge

A few questions arise when considering how to engage in equitable global health research:

1. How can African institutions and physicians benefit from international research collaborations without being exploited?
2. How can advancement of African research capacity and academic careers be prioritized while satisfying the “publish or perish” mandate of HIC universities?
3. How do African scientists and governments coordinate the great influx of HIC academics who view the continent as the next frontier in global health research?

This essay describes some of the important steps for African researchers and academic institutions to consider in managing global health research partnerships in their settings.

## Building Local Research Capacity through HICs and Regional Resources

Few physicians in Africa are trained in research. Of these, some emigrate to HICs where the opportunities for career advancement are greater [16],[17] or are poached by HIC-funded research that is not collaborative or not aligned with national health priorities [10]. Poor funding and lack of protected time for research pursuits are a common complaint by African researchers [18]. A key goal of any global health research collaboration is the transfer of research skills to African partners. HIC institutions can provide their African counterparts with access to distant learning resources such as online libraries, protocol development, statistical expertise, database development, and management. The World Health Organization (WHO) has created HINARI, an initiative that provides free access to thousands of journals for LMIC institutions [19]. The provision of courses in research design, statistical interpretation, and scientific writing can develop skills that are often inadequately developed [20]. A growing number of open courseware continuing education programs make learning research skills more affordable than studying abroad. Furthermore, research capacity in certain African countries such as South Africa is more developed than others; local capacity can be strengthened through regional partnerships. HIC initiatives such as the European & Developing Countries Clinical Trials Partnership (EDCTP) promote South to South collaborations in HIV, tuberculosis, and malaria through pan-African clinical databases and funding of projects [21]. The Training Health Researchers into Vocational Excellence in East Africa (THRiVE) program aims to improve regional research capacity by linking academic institutions from Uganda, Rwanda, Tanzania, and Kenya [22]. Several British universities provide financial and technical support. Another South to South collaboration is the Netherlands–African Partnership for Capacity Development and Clinical Interventions of Poverty-related Diseases (NACCAP), which builds research capacity between several sub-Saharan African academic institutions with support from Dutch partners [23].

## Setting Local Research Agenda

Historically, HIC researchers control funding and therefore dictate research agendas in Africa. Africans need to set their own research priorities. A positive example is the Ubuntu Clinic, which treats HIV and tuberculosis patients in Khayelitsha, South Africa. The clinic has a research committee composed of local physicians from various academic stakeholders who set the research agenda and provide guidance to international researchers [24]. Trusted long term HIC collaborators who understand the context and needs of the region can teach agenda-setting skills and assist in agenda development [14]. Continued dialogue between stakeholders such as local research institutions and their ministries of health will translate local research into action. Regular communication with regional and international health policymakers is needed to understand global health issues and priorities.

## Long Term Collaboration

Long term partnerships facilitate equitable research collaborations. Frequently, personal relationships between individuals can lead to formal partnerships. For example, the Rakai Health Sciences Program in Uganda began in 1987 as a collaboration between two Ugandan physician researchers, Nelson Sewankambo and David Serwadda, and a US colleague, Maria Wawer, on a small community cohort study, which has expanded to a large research program focusing on community prevention trials and studies with many HIC and Ugandan partner institutions [25]. Similarly, the Kenya Medical Research Institute (KEMRI) came into being in 1979 through a personal working relationship between Allan Ronald of the University of Manitoba and Herbert Nsanze of the University of Nairobi and has grown into a large research institution focusing on malaria and HIV/AIDS with national, regional, and international partners [26]. Twinning—a promising new concept in global health—pairs HIC health care institutions or medical schools with counterparts in Africa and other LMICs [27],[28]. HIC collaborators may develop mentorship programs with African counterparts between the twinned institutions. For example, the Africa Centre for Health and Population Studies in South Africa has many equitable HIC collaborations such as partnerships with the Wellcome Trust, Brown University, and the University College London [29].

## Local Coordination and Monitoring

Some HIC academics arrive in Africa, with their own funding, to conduct studies on topics that they have decided on without local input [18]. The large influx of HIC researchers wanting to work in African settings have to be limited to those who genuinely want to collaborate, build local capacity, address locally identified priorities, and treat local counterparts as equals. Distinguishing these collaborators from those who are self-serving is essential and has to be regulated by African leaders. Local coordination and oversight would prevent research duplication and ensure that studies are in line with local policies and priorities. Challenges arise, however, because some African hosts may be enthusiastic about twinning with “prestigious” US universities, which consequently creates a power dynamic that can be inherently unequal and make African institutions reluctant to say “no” to research requests and risk offending their new colleagues [13]. This reluctance to refuse external assistance from HIC partners is also exacerbated by the potential for resource gains. African countries need to engage their ministries of health and academic institutions to provide a monitoring mechanism with a clear set of guidelines. For example, local research committees can be required to screen and approve all projects conducted in the country. Each project is required to demonstrate mutual and equitable benefit such as specific study objectives aligned with local health research priorities, well-defined roles for each collaborator including the unique expertise of HIC partners, and authorship equity for publication planning. A central virtual registry for twinned projects modeled on the ClinicalTrials.gov registry in the United States will increase transparency and accountability in research conduct and be an effective prerequisite for publication [30].

## Establish an Institutional Ethics Review Board

Some research in Africa has exploited local populations [31]–[33]. Many HIC researchers who conduct studies in African countries receive institutional ethics board (IRB) clearance from their own institutions that do not represent the interests of the country where the research will be performed. Local ethics review boards are needed to provide additional oversight and to ensure that all studies comply with International Ethical Standards including protection against exploitation of vulnerable local populations [34]. In Rwanda, HIC researchers must have local partners and all projects must be approved by the Rwanda National Health Research Committee and a local IRB [35]. Memorandums of understanding regarding confidentiality agreements, intellectual property, and data ownership/sharing need to be established *a priori* before any research work begins. Local IRBs should ensure that adherence to policies on intellectual property including data and confidential patient information are respected. One unique consideration is the material transfer of body tissues from Africa to HICs for special tests. Performing these tests locally or at least regionally gives greater African ownership of studies and HIC collaborators need to help build this capacity. Lack of funding, expertise, and appropriate infrastructure to establish appropriate laboratories are current limitations [36]. The WHO and other regional and international collaborations such as the African Field Epidemiology Network and the East Africa Public Health Laboratory Networking Project have projects underway to improve national health laboratory systems [37]–[40].

## Requiring Local Authorship and Dissemination of Results

The goal of any collaboration is to produce high-quality research in order to advance scientific knowledge, clinical care, and to influence evidence-based research and public policy. Publishing ensures transparency, demonstrates accountability for financial support, and allows for establishing metrics of productivity. Collaborative publications need principal investigators from HICs and African partner institutions who were involved in the design, conduct, analysis, and manuscript writing of each individual project. The Ministry of Health in Rwanda requires local authorship on all studies published using local data on the basis of recent experiences with “extractive” research [35]. While this is one step in maintaining local ownership, it is not the only solution and such mandates can be difficult to enforce as token authorship can always be found. Africans are currently under-represented in writing up collaborative work for publication. Studies are needed to quantify authorship equity. Experienced HIC researchers can encourage African co-investigators to present at international conferences, which often offer scholarships to fund travel expenses. Local dissemination of results can be also encouraged through presentations at national medical societies and institutional departmental meetings, thus allowing a wider local audience to benefit from research methodology and results.

## Human Resources for Health and Its Role in Research

One model of a global health partnership is the Human Resources for Health Program in Rwanda. Established in 2012, the HRH program twins 16 US institutions with the Rwandan Ministry of Health and its various medical institutions to “improve the quantity and quality of health professionals in Rwanda” [41]. The program will run for seven years and pairs US physicians and other health care professionals with Rwandan colleagues to transfer clinical, teaching, and research skills. Each US faculty remains in Rwanda for at least one year, allowing time and trust to build with their Rwandan counterparts. This relationship will hopefully be more successful in developing local research capacity and equitable research collaborations compared to previous models of short visiting professorships of a few days or weeks. Skills such as how to set a local research agenda and coordinate other HIC international collaborators will be taught. Pitfalls such as token authorship will be avoided as increased data analysis and write-up capacity are developed.

## Conclusions

Global health partnerships and international research collaborations have enormous potential to improve health care and policy in Africa. The growing field of global health brings a wealth of HIC research experience and funding to African countries. Power imbalances and inequity exist in these processes and for successful research partnerships to occur between HIC and African individuals and institutions, several steps need to be taken for relationships to be both equitable and long term. The transfer of research skills, from HIC collaborators to local partners, is a key objective in every collaboration, in order to build local capacity for investigators to define and coordinate their own research agendas. African countries must take control of their research agendas and coordinate HIC collaborators. Otherwise, African countries risk repeating history and becoming victims of “scientific colonialism” [10].
